# Direct Conversion X-ray Detector with Micron-Scale Pixel Pitch for Edge-Illumination and Propagation-Based X-ray Phase-Contrast Imaging

**DOI:** 10.3390/s22155890

**Published:** 2022-08-07

**Authors:** Abdollah Pil-Ali, Sahar Adnani, Christopher C. Scott, Karim S. Karim

**Affiliations:** 1 Department of Electrical and Computer Engineering, University of Waterloo, 200 University Ave W, Waterloo, ON N2L 3G1, Canada; 2 Centre for Bioengineering and Biotechnology, University of Waterloo, 200 University Ave W, Waterloo, ON N2L 3G1, Canada; 3 KA Imaging, 560 Parkside Dr #3, Waterloo, ON N2L 5Z4, Canada

**Keywords:** X-ray phase-contrast imaging, propagation-based, edge-illumination, coded-aperture, direct conversion detector, amorphous selenium, high-resolution imaging, micron-scale pixel detector

## Abstract

In this work, we investigate the potential of employing a direct conversion integration mode X-ray detector with micron-scale pixels in two different X-ray phase-contrast imaging (XPCi) configurations, propagation-based (PB) and edge illumination (EI). Both PB-XPCi and EI-XPCi implementations are evaluated through a wave optics model—numerically simulated in MATLAB—and are compared based on their contrast, edge-enhancement, visibility, and dose efficiency characteristics. The EI-XPCi configuration, in general, demonstrates higher performance compared to PB-XPCi, considering a setup with the same X-ray source and detector. However, absorption masks quality (thickness of X-ray absorption material) and environmental vibration effect are two potential challenges for EI-XPCi employing a detector with micron-scale pixels. Simulation results confirm that the behavior of an EI-XPCi system employing a high-resolution detector is susceptible to its absorption masks thickness and misalignment. This work demonstrates the potential and feasibility of employing a high-resolution direct conversion detector for phase-contrast imaging applications where higher dose efficiency, higher contrast images, and a more compact imaging system are of interest.

## 1. Introduction

X-ray Phase-Contrast Imaging (XPCi), an emerging modality, has demonstrated early results that are superior to conventional attenuation-based X-ray imaging due to its ability to improve visualization of low-contrast objects. It offers an attractive lower cost and potentially higher throughput, and higher spatial resolution alternative to magnetic resonance imaging (MRI) and other imaging methods in some clinical scenarios. This modality has been clinically evaluated to image breast tissue, cancer specimens, and brain tissue, in addition to characterizing multi-material samples [1,2,3].

Among different techniques developed to realize the XPCi modality, propagation-based (PB), and edge-illumination (EI) can be scaled in a relatively straightforward manner to large area imaging systems, which gives them an advantage over other approaches [4,5]. EI-XPCi, in particular, can provide excellent detail from various objects, which makes it a promising candidate for next-generation commercial X-ray imaging benchtop systems [6]. Although PB-XPCi offers a less complex setup compared to EI-XPCi—which requires a set of two absorption gratings—it possesses mathematically intensive methods to retrieve phase and attenuation information. Although many attempts have been made to advance PB-XPCi information retrieval methods, they vary based on different assumptions and approximations that are made, as well as the complexity of the required input data for different materials, which make them hard to use. Moreover, not all of these information retrieval methods can be applied to applications where polychromatic X-ray sources are employed [7,8,9,10]. On the other hand, information retrieval in EI-XPCi can be performed in a less intensive manner due to a difference in imaging physics [11]. In both PB and EI-XPCi, however, employing a high-resolution X-ray detector can potentially improve the detectability of small feature sizes in objects [12].

High spatial resolution imaging becomes essential and critical for certain medical and non-medical applications. The span of its application varies from microtomography (μ-CT) and mammography—where detecting more details and identifying objects with small feature sizes, such as carcinoma, calcification, or cancerous tumor are crucial and vital, especially at their early stages—to emerging applications in the industry such as semiconductor inspection, machine vision, and material science [13,14,15,16,17,18,19]. Extensive research has been conducted to improve spatial resolution in compact X-ray detectors for energies above 20 keV using a combination of amorphous selenium (a-Se) photoconductors and complementary metal-oxide semiconductor (CMOS) readout technology with micron-scale pixels [20,21,22].

Imaging at higher resolutions, higher X-ray energies, and lower exposures are three main and highly interdependent criteria for enhancing any XPCi system, but absorption mask gratings are central to improving an EI-XPCi system. Theoretically, employing high-resolution absorption gratings along with a high-resolution detector, and considering a compatible microfocus X-ray source, should improve EI-XPCi performance. However, such a high-performance EI-XPCi system has not yet been realized due to technological challenges. Two main bottlenecks that impede the realization of such a high-resolution EI-XPCi system are: (1) fabrication of high-resolution (fine pitch) absorption gratings compatible with the small pixel size of such high-resolution detectors, and (2) mechanical vibration sensitivity of a high-resolution EI-XPCi system [23].

In this work, we first introduce a framework by which both PB-XPCi and EI-XPCi can be modelled and numerically simulated; we verify the numerical simulation results using experimental data to validate the model. This model, at a system level, takes into account the effect of source, detector, and pixel blurring; in addition, the thickness and mechanical vibration of the absorption gratings in the final image. By utilizing this model, we analyze PB-XPCi and EI-XPCi methods and evaluate their performance in terms of contrast, edge-enhancement, visibility, and dose efficiency characteristics to explain how EI-XPCi can outperform PB-XPCi with the same X-ray source and detector. This higher performance is due to the fact that EI-XPCi contains less background attenuation signal than PB-XPCi—which can be interpreted as an unwanted signal; thus, EI-XPCi produces a phase image with higher contrast compared to PB-XPCi. Despite EI-XPCi’s superiority over PB-XPCi, simulation results reveal that the absorption masks thickness variation and mechanical vibration can significantly affect the behavior of a high-resolution EI-XPCi system. Next, we present existing PB-XPCi and EI-XPCi systems and demonstrate what type of technology is currently being used in each system. A BrillianSe™ prototype is introduced as a direct conversion a-Se/CMOS-based high-resolution integration mode X-ray area detector with a pixel pitch equal to 7.8 μm [24]. Ultimately, we show how the X-ray absorption grating fabrication industry is one step behind the high-resolution detector technology, particularly in EI-XPCi systems.

## 2. Methods

### 2.1. Theory and Model

**Figure 1:**
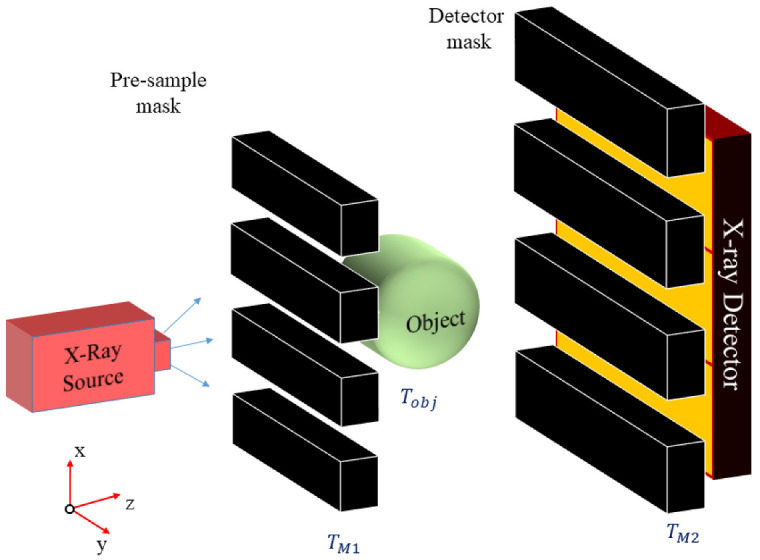
Components and geometry of the EI-XPCi (as well as PB-XPCi) system model.

Both PB-XPCi and EI-XPCi systems can be mathematically modelled based on electromagnetic wave transmission through objects and propagation in free-space. Figure 1 illustrates the components and geometry of an EI-XPCi system model. It consists of an X-ray source, a pre-sample absorption mask, an object, a detector absorption mask, and an X-ray detector. It is assumed that the pre-sample mask and detector mask are at the object plane and detector plane, respectively. Following the Fresnel–Kirchhoff integral to describe the propagation of X-rays between source and object, as well as object and detector, the expected signal intensity at position (x,y) registered at the detector plane—$I_{d}\left(x,y\right)$—can be expressed through Equations (1)–(6):

(1) $$\begin{array}{c} \;\;\;\;\;\;\;\;\;\;\;\;\;I_{d}\left(x,y\right)=\int_{}^{}\int_{}^{}EgN\left(x,y,E\right)\times \\ F^{-1}\left\{MTF\times F\left\{F^{-1}\left\{F\left\{S_{r}\left(x,y\right)\right\}\times F\left\{I_{p}\left(x,y,E\right)\right\}\right\}\times |T_{M2}\left(x,y\right){|}^{2}\right\}\right\}dEdxdy\;, \end{array}$$

(2) $$N\left(x,y,E\right)=\Phi \left(E\right)tw^{2}\eta_{d}\left(E\right)\;,$$

(3) $$MTF=MTF_{s}\times MTF_{d}\times MTF_{p}\;,$$

(4) $$I_{p}=|F^{-1}\left\{F\left\{H_{1}\left(x,y,E\right)\times T_{M1}\left(x,y\right)\times T_{obj}\left(x,y,E\right)\right\}\times F\left\{H_{2}\left(x,y,E\right)\right\}\right\}{|}^{2}\;,$$

(5) $$H\left(x,y,E\right)=\frac{e^{ikz}}{i\lambda z}e^{\left(ik\frac{x^{2}+y^{2}}{2z}\right)}\;,$$

(6) $$T_{\mathrm{obj}}\left(x,y,E\right)=e^{\left(i\phi (x,y,E)-M(x,y,E)\right)}\;,$$

where *E* is the photon energy; *g* is the detector gain; N(x,y,E) is the number of X-ray photons; MTF is the combined modulation transfer function of source, detector, and pixel; $S_{r}\left(x,y\right)$ is the re-scaled source intensity distribution on the detector plane; $I_{p}\left(x,y,E\right)$ is the intensity measured at a pixel; Φ(E) is the photon flux (photons per time and area) in the detector plane when no object is present; *t* is the duration of exposure time; $w^{2}$ is the pixel area; $\eta_{d}\left(E\right)$ is the detector absorption efficiency; $H_{1}\left(x,y,E\right)$ is the Fresnel propagator between source and object plane; $H_{2}\left(x,y,E\right)$ is the Fresnel propagator between the object plane and detector plane; $T_{M1}$ is the pre-sample mask transmission function and $T_{M2}$ is the detector mask transmission function, where both are equal to one within the apertures and are equal to zero in the absorbing septa; $T_{obj}$ is the object transmission function; λ is the wavelength of a monochromatic wave; *k* is the wave number in the *z*-propagation-direction; as well as ϕ(x,y,E) is the phase shift and M(x,y,E) is the linear amplitude attenuation, both calculated from the complex refractive index of the object [25,26].

Considering that the object of interest is described by its complex refractive index through Equation (7):

(7) n(x,y,z)=1−δ(x,y,z)+iβ(x,y,z),

where δ (phase coefficient) and β (absorption coefficient) are related to the refraction and absorption of the object, respectively. If the material composition of the object is known, one can calculate δ and β using Equations (8) and (9):

(8) $$\delta =\frac{r_{0}h^{2}c^{2}}{2\pi E^{2}}\sum_{Z}n_{Z}f_{1Z}^{0}\left(E\right),$$

(9) $$\beta =\mu \frac{hc}{4\pi E},$$

with $r_{0}$ being the Thomson scattering length or the classical electron radius, *h* the Planck’s constant, *c* the speed of light, and *E* the photon energy; $n_{Z}$ is the atomic density, *Z* is the atomic number with a summation over all present elements in the object material composition, $f_{1Z}^{0}$ is the forward scattering factor that can be considered equal to *Z* in this case, and μ is the linear attenuation coefficient [26]. Using Equations (8) and (9), we can calculate ϕ(x,y,E) and M(x,y,E) through Equations (10) and (11):

(10) $$\phi \left(x,y,E\right)=k\int_{-\infty }^{z}\delta \left(x,y,z\right)dz,$$

(11) $$M\left(x,y,E\right)=2k\int_{-\infty }^{z}\beta \left(x,y,z\right)dz.$$

The required thickness of a material to stop an X-ray photon can be calculated using exponential attenuation law through Equation (12):

(12) $$\frac{I}{I_{0}}=e^{-(\frac{\mu }{\rho })x}.$$

**Figure 2:**
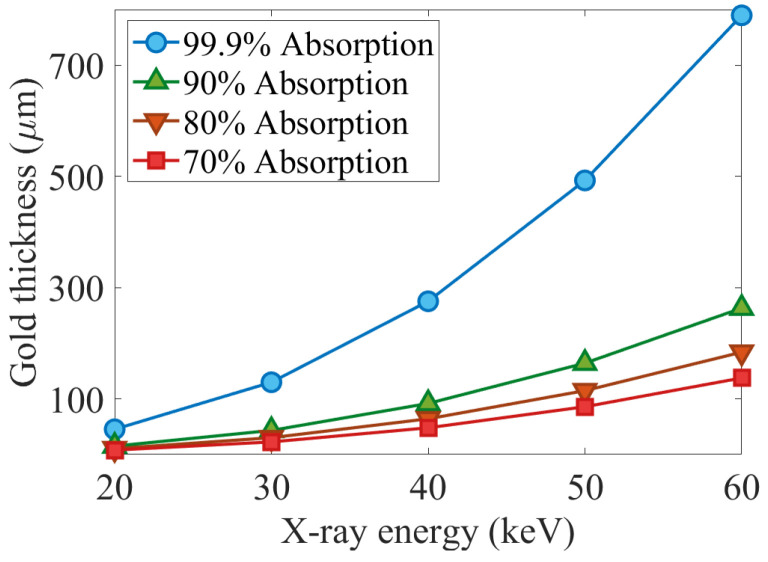
Required mask thickness (thickness of absorption part, which is gold here) to stop the X-ray energy.

It is worth mentioning that the mass thickness is defined as the mass per unit area, and is obtained by multiplying the thickness $x_{t}$ by the density ρ, i.e., $x=\rho x_{t}$. Gold is selected as the X-ray absorber for absorption masks design since it is widely used in X-ray mask fabrication. Figure 2 illustrates the required thickness of gold (for an absorption mask) versus X-ray energy so the X-ray can be stopped in an absorption mask.

Environmental vibration has a negative effect on the EI-XPCi system. In this work, environmental vibration is translated into mechanical displacement in the mask misalignment. It should be noted that, for the sake of simplicity, this vibration is modelled through mechanical displacement along only the x-direction of the pre-sample mask, since line gratings (Figure 1) are assumed for this study. It is assumed that masks misalignment associated with mechanical vibration is within a range of two micrometres. We have simulated different scenarios in which mask misalignment varies within this range for both a high-resolution EI-XPCi (pixel size equal to 7.8 μm to match the prototype detector) and an EI-XPCi system with a bigger pixel pitch (39 μm, i.e., 5×5 pixel binning) to highlight the effect of vibration on a high-resolution EI-XPCi system.

### 2.2. Simulation and Verification

**Figure 3:**
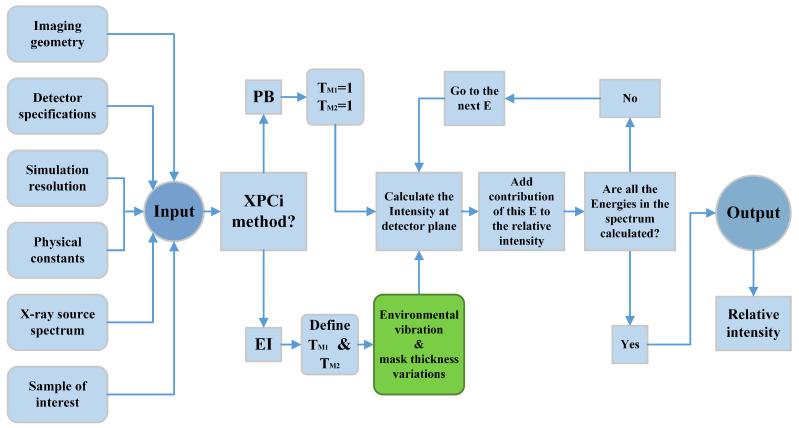
Algorithm used to simulate the model for both PB-XPCi and EI-XPCi configurations in MATLAB^®^. The green block represents where absorption mask thickness variation and mechanical vibration are defined in the model.

The model explained here works well for both monochromatic as well as polychromatic X-ray sources, where, in the latter scenario, the intensity registered at the detector is calculated by integration over each individual X-ray energy component contributed to the polychromatic source energy spectrum. This model is numerically simulated in MATLAB^®^ following the algorithm shown in Figure 3. It should be noted that we employed the *spektr* toolset to simulate the X-ray spectrum and mass-energy attenuation coefficient—the data of which are retrieved from NIST [27].

**Figure 4:**
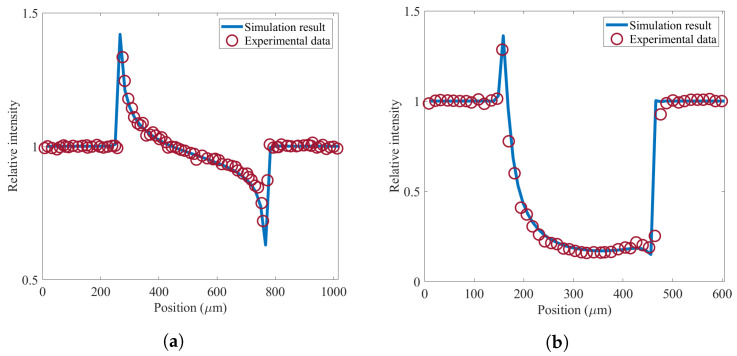
Comparison between the simulation result and experimental data extracted from [25] for (**a**) a PEEK wire, and (**b**) a Titanium wire. The solid blue line represents the simulation result, and the red circles demonstrate experimental data.

To verify the model, simulation results are compared with experimental data from [25] (using a quasi-monochromatic X-ray beam with an energy of 20 keV), which are illustrated in Figure 4. Simulation is done assuming a quasi-monochromatic Gaussian distributed X-ray source with FWHM = 80 μm at 40 kV${}_{p}$ (equal to 20 keV); source to sample distance of 20 m and sample to detector distance of 0.55 m; as well as 50% illumination fraction (for EI-XPCi setup) for two different samples: polyetheretherketone (PEEK) wire with a diameter equal to 460 μm, and titanium wire with a diameter equal to 250 μm. PEEK is selected as an almost transparent object to the X-ray energy considered for this comparison, hence it reveals an intensity profile that is mainly due to phase shift rather than attenuation contrast; and Titanium is chosen as an object which presents a strong absorption, thus a wide range of conditions is covered to verify the model.

## 3. Comparison of High-Resolution PB-XPCi and EI-XPCi

**Figure 5:**
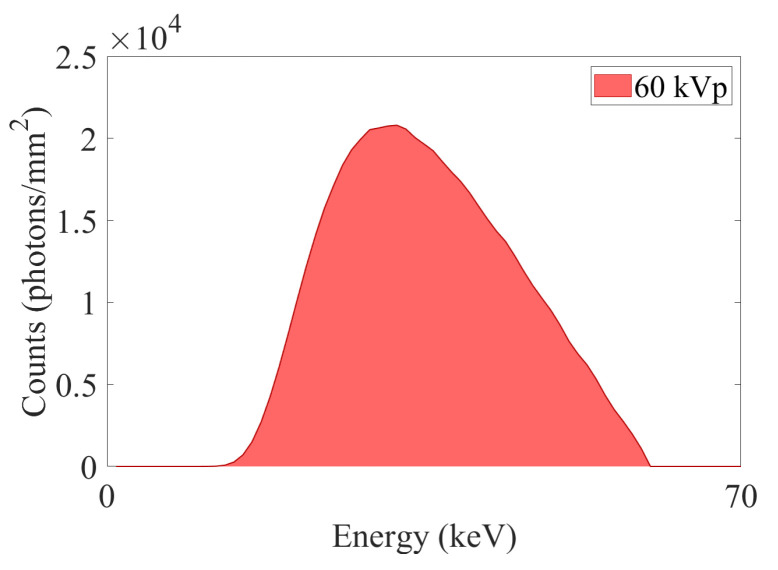
X-ray spectrum of tungsten target employed from the *spektr* toolset in MATLAB^®^ for simulation. The spectrum is filtered by 0.5 mm aluminum (Al).

The model explained in the previous section is employed to investigate the PB-XPCi and EI-XPCi systems and to evaluate them, where we have considered a polychromatic microfocus X-ray source working at 60 kV${}_{p}$ with a spot size equal to 9 μm (except for studying the effect of $MTF_{d}$ on the imaging performance where we consider a spot size equal to 2 μm) and a detector with pixel size and pitch equal to 7.8 μm for a PEEK wire with a diameter equal to 585 μm as the object of interest. Figure 5 illustrates the X-ray spectrum employed in this work to simulate the model.

Contrast, edge-enhancement, and visibility characteristics of PB-XPCi and EI-XPCi are evaluated and compared using Equations (13)–(15); dose efficiency, however, is considered as a relative measure to compare how much of the incident X-ray is registered at the object:

(13) $$\mathrm{Contrast}=\frac{I_{max}-I_{min}}{I_{background}},$$

(14) $$\mathrm{Edge-Enhancement}\;\left(\%\right)=\frac{I_{max}-I_{background}}{I_{background}}\;\times \;100,$$

(15) $$\mathrm{Visibility}=\frac{I_{max}-I_{min}}{I_{max}+I_{min}},$$

where $I_{max}$, $I_{min}$, and $I_{background}$ are the maximum, minimum, and background signals registered at the detector, respectively.

### 3.1. Direct vs. Indirect Conversion Detector

**Figure 6:**
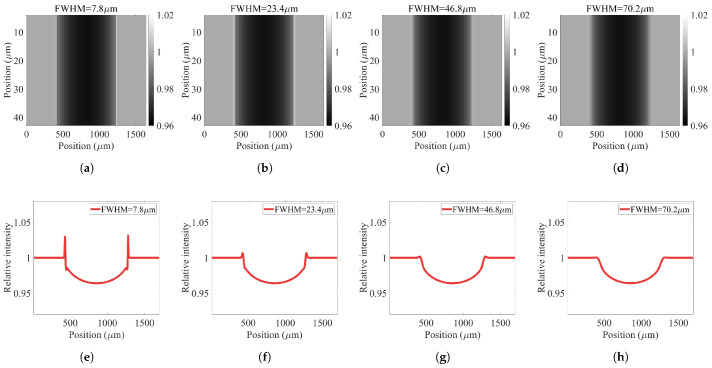
The effect of detector modulation transfer function ($MTF_{d}$) on image quality and relative intensity. (**a**,**e**) represent a direct conversion detector with a pixel size equal to 7.8 μm. (**a**,**e**) represent a direct conversion detector with a pixel size equal to 7.8 μm. (**b**,**f**) represent a direct conversion detector with a pixel size equal to 23.4 μm. (**c**,**g**) represent a direct conversion detector with a pixel size equal to 46.8 μm. (**d**,**h**) represent a direct conversion detector with a pixel size equal to 70.2 μm. Simulation is done for a PEEK wire with 585 μm in diameter using a polychromatic microfocus X-ray source working at 60 kV${}_{p}$ with a spot size equal to 2 μm to only study and highlight the effect of $MTF_{d}$ on the system performance.

To highlight the advantage of employing a high-resolution direct conversion detector and its effect on image quality, we simulated and compared a direct conversion detector with an indirect conversion detector. The indirect conversion detector is modelled by modifying its $MTF_{d}$. To study only the effect of $MTF_{d}$ on the imaging performance, simulation is performed considering $MTF_{p}$ and $MTF_{s}$ with smaller point spread function (PSF) values than the $MTF_{d}$. Simulation is done for different values of full width at half maximum (FWHM) of a detector PSF to illustrate the effect of $MTF_{d}$ on the image quality. Figure 6 demonstrates the impact of $MTF_{d}$, which is represented here as different values of the FWHM of the detector PSF. As the detector PSF increases, the imaging resolution decreases, leading to losing the object information in the registered image. For the same detector pixel size, indirect conversion detectors suffer from greater detector PSF, which leads to resolution degradation. However, direct conversion detectors benefit from smaller PSF, making them superior for high-resolution imaging tasks [24]. From a qualitative imaging perspective [28], direct conversion detectors can reveal more details, which is beneficial for XPCi applications where the edge enhancement of a pure phase object plays a critical role (Figure 6a), compared to indirect conversion detectors where details are lost ((Figure 6d). Moreover, for quantitative studies, direct conversion detectors make it easier to extract more information from an image when phase-contrast properties are present (compare Figure 6e with Figure 6h).

**Figure 7:**
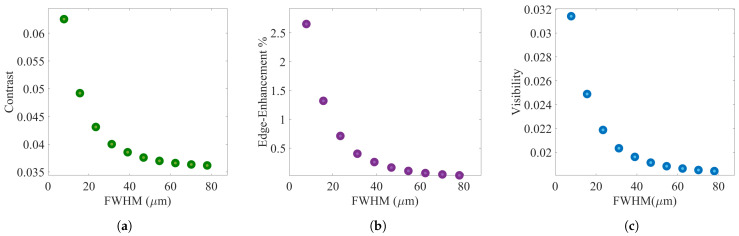
(**a**–**c**) Simulation results of contrast, edge-enhancement, and visibility for a PB-XPCi system carried out for a PEEK wire with 585 μm in diameter as a function of different detector modulation transfer function values ($MTF_{d}$).

Simulation results confirm that, increasing the FWHM of the detector PSF lowers the contrast, edge-enhancement, and visibility of a PB-XPCi system (the same results can be expected for an EI-XPCi system), as illustrated in Figure 7. The highest value in the three graphs in Figure 7 (at FWHM=7.8μm) corresponds to a direct conversion detector with a pixel size equal to 7.8 μm, and higher FWHM values in Figure 7 represent an indirect conversion detector (with the same pixel size).

### 3.2. PB-XPCi

**Figure 8:**
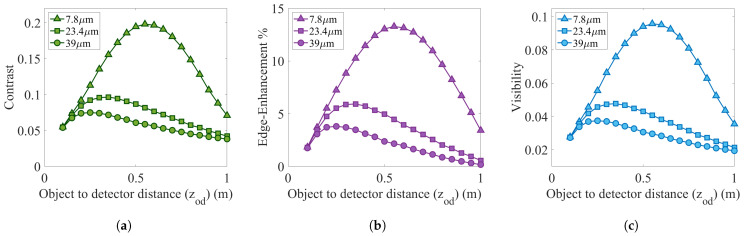
Simulation results of the behavior of contrast, edge-enhancement, and visibility of a PB-XPCi system versus the object-to-detector distance, carried out for a PEEK wire with 585 μm in diameter.

With $z_{so}$, $z_{od}$, and $z_{sd}$ being the source-to-object, object-to-detector, and source-to-detector distances, investigation of contrast, edge-enhancement, and visibility characteristics are carried out for PB-XPCi as a function of $z_{od}$, considering the fact that $z_{sd}$ is kept constant—105 cm. In other words, PB-XPCi is evaluated at different magnification scenarios (between 1.05 to 21) when $z_{od}$ is changing between 5 cm to 100 cm, and $z_{sd}$ is always kept constant—equal to 105 cm. Figure 8a–c illustrate the contrast, edge-enhancement, and visibility for a PB-XPCi system. In order to highlight the role of a high-resolution detector in the imaging performance, three different pixel sizes of 7.8 μm, 23.4 μm, and 39 μm are simulated all through the same imaging system condition. Reducing the pixel size results in an enhancement in the detected signal, which leads to higher contrast, edge enhancement, and visibility. Since PB-XPCi systems require high spatial coherence X-ray sources to reveal the phase-contrast effect, phase shift drops for lower spatial coherence components. This is noticeable in Figure 8a–c, where increasing in $z_{od}$—in other words higher magnifications—results in lower contrast, less edge-enhancement, and less visibility. On the other hand, contrast, edge-enhancement, and visibility experience lower values when the object is close to the detector—at lower $z_{od}$, since the phase effect has not yet been evolved due to a lower propagation distance between the object and detector.

### 3.3. EI-XPCi

**Figure 9:**
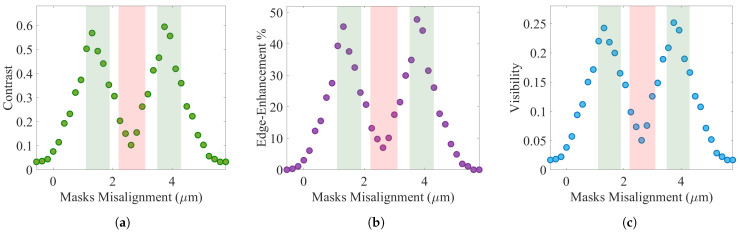
Simulation results of the behavior of contrast, edge-enhancement, and visibility of an EI-XPCi system versus masks misalignment carried out for a PEEK wire with 585 μm in diameter.

An EI-XPCi system performance relies on parameters such as its masks dimensions, detector pixel size, as well as X-ray source energy; thus, $z_{so}$ and $z_{od}$ should be selected and optimized based on the system configuration, and they should be kept fixed, since altering their values affect the system behavior. As a result of that, contrast, edge-enhancement, and visibility are investigated as a function of masks misalignment—which results in different illumination fractions—for the EI-XPCi system. Investigation of the EI-XPCi system is done based on a 7.8 μm pixel size detector; a pre-sample mask with 1.56 μm aperture and 6.24 μm period; a detector mask with 1.17 μm aperture and 7.8 μm period; $z_{so}$ of 18 cm; and $z_{od}$ equal to 4.5 cm. The aperture and period of pre-sample and detector masks are designed based on the X-ray source specifications, distances, and pixel size. Although the detector mask aperture can be greater than the aperture of the pre-sample mask, we designed it to be smaller to minimize the effect of source blurring and to cover different scenarios in changing the masks misalignment. An EI-XPCi system behavior highly depends on where the beamlets, which exited the pre-sample mask, land on the detector mask. Considering a beamlet to exemplify the situation, three different scenarios could occur: (1) the whole beamlet impinges upon the absorption part of the detector mask (Figure 1), therefore nothing is detected; (2) a portion of the beamlet is absorbed by detector mask and the rest is registered by the pixel—this is the definition of edge-illumination; (3) the beamlet is fully landed within the detector mask aperture. Since only the second scenario leads to edge-illumination behavior, this is worth mentioning that, for mask misalignment, there is a point (or a number of points) at which the system operates to its highest potential for image acquisition. Figure 9a–c illustrate the contrast, edge-enhancement, and visibility of the EI-XPCi system when masks are misaligned over the span of one pre-sample mask period to demonstrate the behavior of the system. The two vertical green bars in Figure 9a–c highlight the regions at which EI-XPCi system reveals the highest contrast (also edge-enhancement and visibility). The middle vertical red bar in Figure 9a–c represents the first scenario where the beamlets of X-ray are fully absorbed by the detector mask, and no signal is detected (without any object present). On the contrary, the far left and far right plateaus are where approximately all of the X-rays are registered by the detector (without any object present).

Dose efficiency, however, is considered as the amount of dose the object receives. The optimum condition would be illuminating the object at the lowest possible dose and achieving the highest contrast in the image simultaneously. In order to investigate these two systems in terms of dose efficiency, we need to review how each system does work. In the PB-XPCi system, nothing blocks the incident X-ray when it propagates toward the object, thus the object is fully illuminated. This is the highest amount of radiation dose an object can receive in any X-ray imaging system. On the other hand, in the EI-XPCi approach, there is an absorption mask right before the object (pre-sample mask), which translates the incident X-ray into smaller beamlets. This translation decreases the delivered X-ray dose at the object. Considering a pre-sample mask with 1.56 μm aperture and 6.24 μm period, only 25% of the incident X-ray will be employed for image acquisition, which is a higher dose efficiency—less delivered radiation dose by the object—compared to the PB-XPCi system. However, it should be mentioned that reducing the dose exposure results in a lower signal-to-noise (SNR) ratio, which in some cases can be later compensated by longer imaging time (or more powerful X-ray sources) to have more X-ray photons. Therefore, a considerable reduction in the delivered dose at the object can be achieved through the EI-XPCi system [2]. It is worth mentioning that, from an image perspective, contrast is more important than SNR since that is how features can be distinguished in the image. In the EI-XPCi system, for the same dose efficiency or as defined in this work, dose absorbed by the object (achieved by increasing exposure time to compensate for signal loss in the pre-sample mask), one would expect higher contrast when compared to PB-XPCi using the system described in this manuscript.

The best system, comprehensively, is the one in which both contrast (also edge-enhancement and visibility) and dose efficiency are high. According to simulation results carried out here, the EI-XPCi system outperforms the PB-XPCi one in contrast, edge-enhancement, visibility, and dose efficiency characteristics to a great extent.

### 3.4. Effect of Absorption Mask Thickness Variation and Mechanical Vibration in a High-Resolution EI-XPCi System

**Figure 10:**
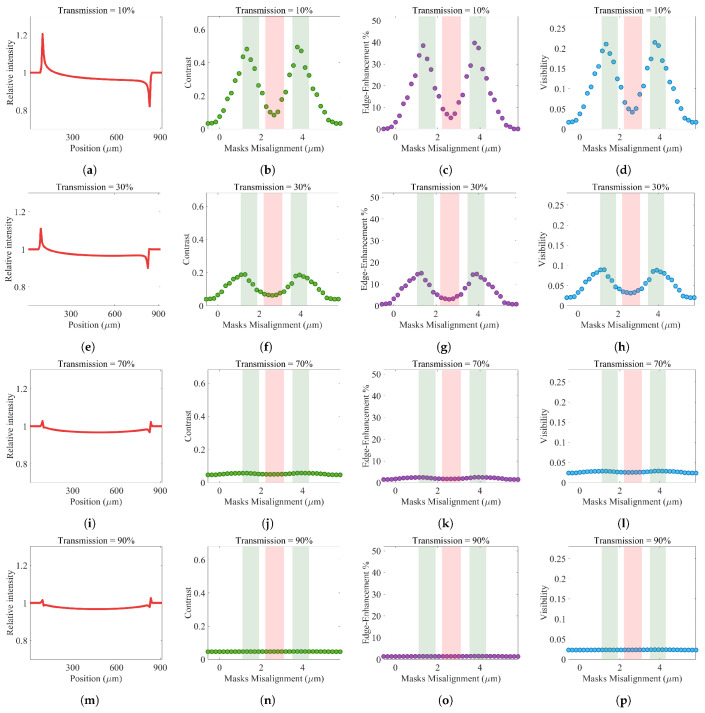
The effect of detector mask thickness variation on the performance of a high-resolution EI-XPCi system. As beamlets’ transmission through the absorption mask increases, the system shifts from EI to PB behavior.

Figure 10 illustrates simulation results of the impact of detector mask thickness variation on the behavior of a high-resolution direct conversion detector (with 7.8 μm pixel size) for an EI-XPCi system. It shows that transmissions up to 30% of the incident X-ray through absorption masks will not considerably change the behavior of the EI-XPCi system (Figure 10a–h). However, transmissions of more than 40% of the incident X-ray through absorption masks result in the EI-XPCi system experiencing a slight change in its behavior—shifting from EI to PB behavior. Transmissions of more than 70% of the incident X-ray through absorption masks highlight the behavior of a PB-XPCi system rather than an EI-XPCi one (Figure 10i–p). The gradual shift from EI to PB behavior, as detector mask thickness decreases, is associated with the increased background signal. The detector mask blocks a portion of incoming beamlets to realize an EI-XPCi system, where any imperfections with the detector mask would result in the EI-XPCi system performance loss. The same shift in behavior, from EI to PB, exists for imperfections in the pre-sample mask, where, in this scenario, impure beamlets generated from an imperfect pre-sample mask result in losing the EI behavior, which is detecting individual beamlet changes.

**Figure 11:**
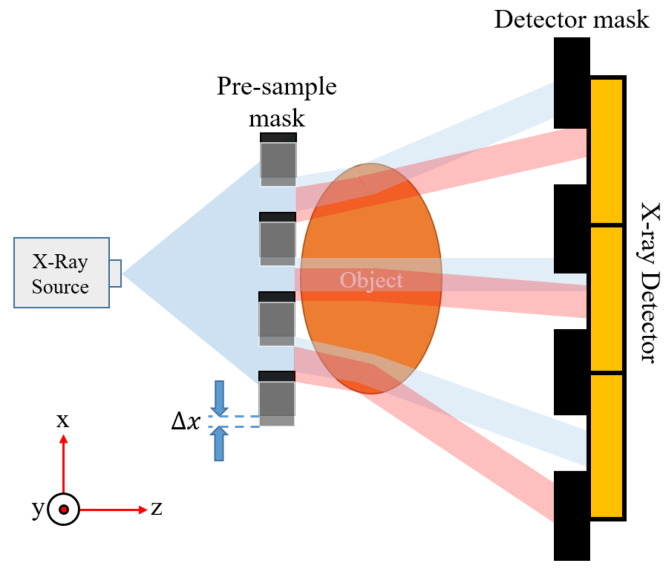
Environmental vibration modelled through mechanical displacement which causes misalignment between pre-sample and detector mask in an EI-XPCi system for high-resolution imaging application.

Figure 11 displays how environmental vibration is modelled through mechanical displacement. For the sake of simplicity, we only considered the pre-sample mask displacement along the x-direction. Displacements along the y-direction do not change the signal intensity as they are parallel with line gratings. In addition, any displacement along the *z*-direction does not change the signal intensity significantly as it hardly changes the shape of beamlets (considering the 2 micrometres range of displacement). The red beamlets (in Figure 11) illustrate the situation in which the pre-sample mask is shifted due to environmental vibration, where the beamlets’ position impinging upon the detector is changed. To study the effect of mechanical vibration on the performance of an EI-XPCi system, we considered two different systems:

1. A 39 μm pixel size system with a pre-sample mask with 4.68 μm and 31.2 μm aperture and period; a detector mask with 9.75 μm and 39 μm aperture and period; $z_{so}$ of 160 cm; and $z_{od}$ equal to 40 cm.
2. A 7.8 μm pixel size system with a pre-sample mask with 1.56 μm and 6.24 μm aperture and period; a detector mask with 1.17 μm and 7.8 μm aperture and period; $z_{so}$ of 18 cm; and $z_{od}$ equal of 4.5 cm.

**Figure 12:**
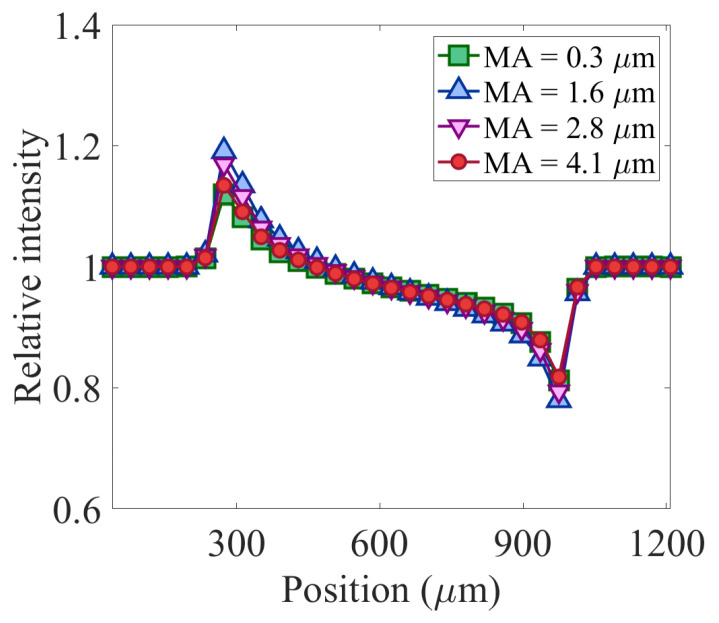
Illustration of simulation results of the effect of environmental vibration in the range of 2 μm on the performance of an EI-XPCi system with pixel size equal to 39 μm × 39 μm.

**Figure 13:**
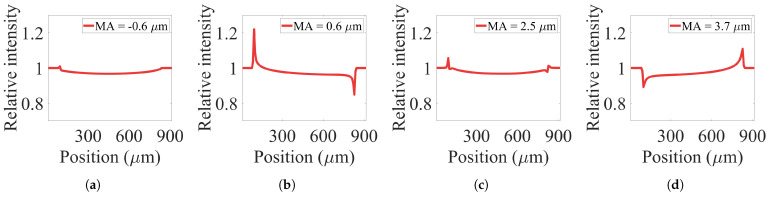
(**a**–**d**) Illustration of simulation results of the effect of environmental vibration on the performance of an EI-XPCi system with pixel size equal to 7.8 μm × 7.8 μm. The behavior of the system dramatically changes even with less than one micrometre shift in the mask misalignment.

Simulation results confirm that high-resolution EI-XPCi systems are sensitive to environmental vibration, where their behavior changes dramatically when mask misalignment swings even in the range of 2 micrometres. However, EI-XPCi systems with bigger pixel sizes are not influenced noticeably by this misalignment. Figure 12 illustrates how environmental vibration within the range of two micrometres does not have a noticeable effect on the behavior of the system with 39 μm pixel size. On the other hand, as Figure 13 shows, the same environmental vibration in higher resolution detectors (in this case, a system with 7.8 μm pixel size) significantly swings the system behavior between PB and EI modes.

This study highlights that, as we move towards higher resolution systems, mechanical vibration can become a significant issue in the system performance, which was not a contributing factor in the performance of lower resolution systems. In general, if proper design practices are followed, environmental vibration can be minimized, and thus it is not a common problem in today’s large fixed X-ray inspection systems. However, if one is trying to design a benchtop or mobile XPCi system or, alternately, placing such a system on an active industrial factory floor, then vibration can become a concern. As a result, higher precision mechanical stages (or optical tables) are required to dampen the environmental vibration. One solution is to use a feedback mechanism to minimize the effect of environmental vibration in post-processing steps. For example, by knowing the intensity transfer function (illumination curve) of the EI-XPCi system and the background signal, any vibration in the background can be translated into the change of intensity, which can be later corrected to lock the masks in position.

## 4. Current XPCi Technologies

### 4.1. Propagation-Based X-ray Phase-Contrast Imaging System

**Figure 14:**
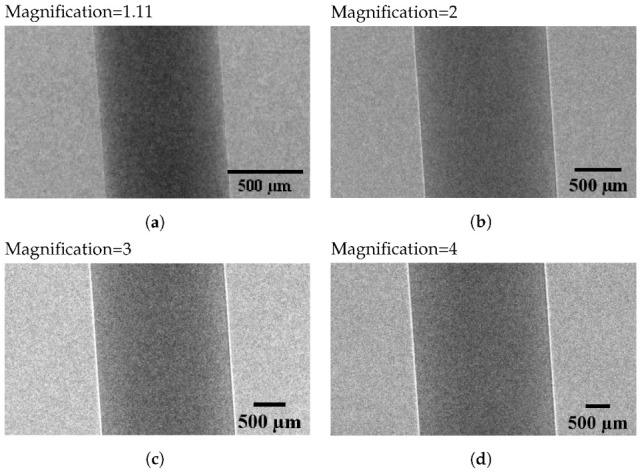

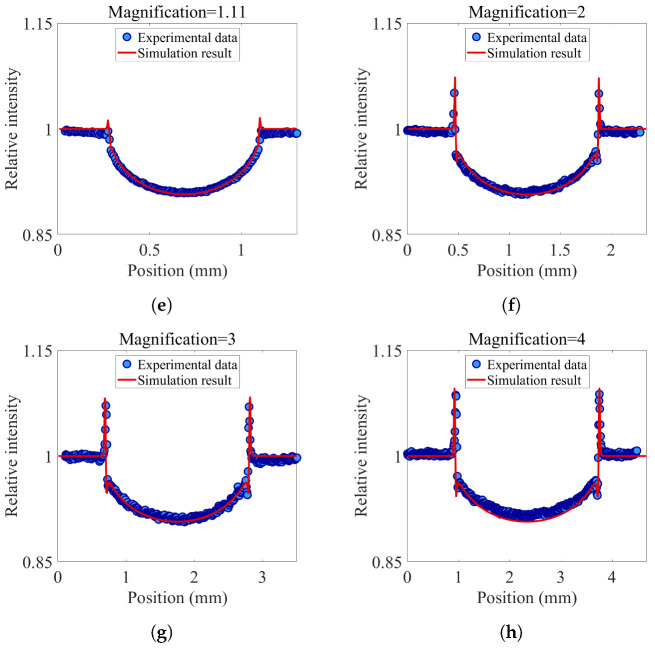
Edge-enhancement effect of PB-XPCi for a 0.7 mm PTFE rod at different magnifications from 1.1 to 4.

Here, we present BrillianSe™, an amorphous selenium (a-Se) integration mode X-ray detector, which employs a hybrid a-Se/CMOS detector technology. This high-resolution detector was initially developed at the University of Waterloo, and is now commercialized by a University of Waterloo spin-off company, KA Imaging Inc. [21,22,29]. The a-Se photoconductor possesses a high intrinsic spatial resolution and is utilized in this X-ray detector in the direct conversion mode. The 7.8 μm pixel pitch BrillianSe™ prototype used in this work has micron-scale resolution and high detection efficiency for energies >20 keV. We used this detector in a PB-XPCi configuration to demonstrate the potential of this high-resolution X-ray detector. The edge-enhancement effect in PB-XPCi at different geometric magnifications for a Polytetrafluoroethylene (PTFE) rod (with a thickness of 0.7 mm) employing a prototype version of BrillianSe™ X-ray detector—1000 × 1000 pixels with 7.8 μm pixel pitch; a tungsten target Thermo Scientific™ PXS5-927 Microfocus X-ray Source (from Thermo Fisher Scentific Inc., Waltham, MA, USA) working at 60 kV${}_{p}$, 134 μA, and focal spot size of 9 μm; as well as the source to object distance of $z_{so}$ = 18 cm is demonstrated in Figure 14. As we increase the distance between the object and the detector (by keeping the source to object distance constant and equal to $z_{so}$ = 18 cm), the edges of the object—where the refractive index changes abruptly—become more detectable. Figure 14a–d illustrate phase-contrast images of the PTFE rod at different geometric magnifications, and Figure 14e–h demonstrate cross-sectional view of the relative intensity profile at different object to detector distances, correspondingly. A good agreement between the simulated model and experimental results can be found here.

**Figure 15:**
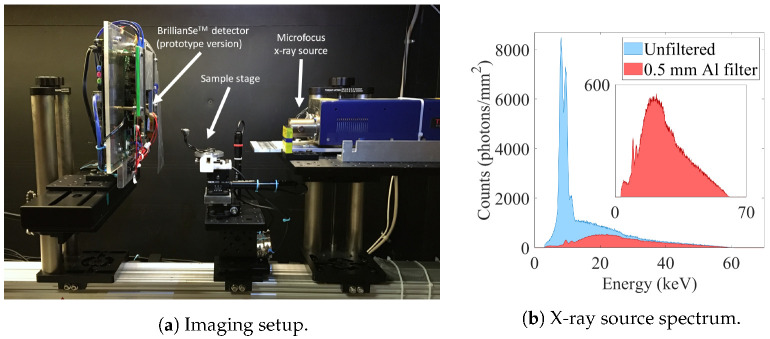
(**a**) The experimental setup used for PB-XPCi imaging with the tungsten target Thermo Scientific™ PXS5-927 Microfocus X-ray Source, sample stage, and a prototype version of the BrillianSe™ X-ray detector; (**b**) the spectrum of the source used for imaging—both the unfiltered and 0.5 mm Al filtered spectrum.

Figure 15a displays the imaging setup and configuration used to perform the PB-XPCi. It should be emphasized that, using a high-resolution X-ray detector like BrillianSe™ not only helps us reveal smaller feature sizes and more information, but makes the final XPCi setup more compact as well. Compactness of an XPCi setup is an advantage when it comes to benchtop and portable imaging systems, such as in intraoperative specimen imaging applications. It should be noted that utilizing such a high-resolution detector to its full potential for higher magnification imaging tasks requires X-ray sources with a finer focal spot, thus the source blurring does not play the limiting factor in the image formation.

**Figure 16:**
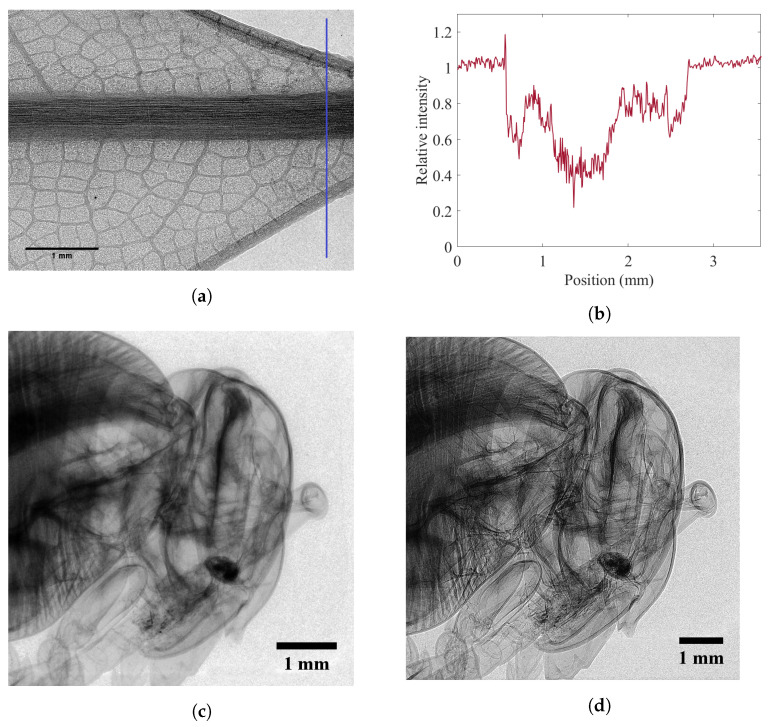
(**a**) Phase-contrast image of a dried bay leaf using a prototype version of the BrillianSe™ X-ray detector and a microfocus source at 60 kV${}_{p}$, $z_{so}$ = 18 cm, and $z_{od}$ = 8 cm; (**b**) profile of relative intensity along the vertical cross-section (blue line); (**c**) X-ray attenuation image of a bee head imaged at 60 kV${}_{p}$, $z_{so}$ = 18 cm, and $z_{od}$ = 1 cm; (**d**) X-ray phase-contrast image of the same bee head imaged at 60 kV${}_{p}$, $z_{so}$ = 18 cm, and $z_{od}$ = 8 cm.

The tungsten target X-ray spectrum used for imaging purposes in this work is illustrated in Figure 15b. The spectrum without any filtration has multiple characteristic lines at around 10 keV, which was filtered using a 0.5 mm aluminum (Al) filter (the inset graph in Figure 15d shows the zoomed-in version of the filtered spectrum). Figure 16a displays the phase-contrast image of a dried bay leaf imaged at 60 kV${}_{p}$, $z_{so}$ = 18 cm, and $z_{od}$ = 8 cm. It is worth mentioning that darker regions in the image displayed in Figure 16a correspond to lower X-ray transmission through the object. Figure 16a reveals the bay leaf’s midrib, margins, veins, and lamina where feature sizes as small as 15–20 μm are clearly visible. Profile of relative intensity along the cross-section (blue line), in Figure 16a, is illustrated in Figure 16b, which indicates phase-contrast peaks at the boundaries of bay leaf’s margins, midrib, and also different parts within the lamina. A bee head is also imaged through the same setup in two different scenarios, first when the object is close to the detector ($z_{od}$ = 1 cm), which corresponds to the attenuation image (Figure 16c), and then when the detector is put further away from the object ($z_{od}$ = 8 cm) (Figure 16d) which leads to a phase-contrast image. There is a small difference in resolution, roughly 7.8 μm for the attenuation image and 5.5 μm for the phase-contrast image—due to the magnification in the latter image. However, the slightly better resolution is insufficient to explain the significantly improved contrast in the phase-contrast image, which is due to phase contrast refraction effects. The clear edge-enhancement around the boundaries of the bee organs observable in the phase-contrast image is thanks to the phase effect and the high-resolution detector employed, all in a compact system.

### 4.2. Edge-Illumination X-ray Phase-Contrast Imaging System

Today’s existing EI-XPCi systems employ mostly X-ray detectors with pixel sizes in the range of 50 to 100 μm. Table 1 is a short list of EI-XPCi systems that are currently being used. Because of the detector pixel sizes used in these setups, they usually take up at least 1.8 m of space. One of the obstacles in the way of developing EI-XPCi systems and upgrading them to smaller pixel size detectors is the fabrication of absorption masks that could match a high-resolution X-ray detector pixel size in a compact setup. Shorter distances and more compact setups impose higher requirements on the absorption masks specifications. Some of the main challenges in the fabrication of absorption masks, which are periodic micro-structures of an X-ray absorber with high-aspect ratios, for high-resolution detectors are to achieve:

1. High-aspect ratio structures (in the range of 40:1, structural width in the range of a few micrometres and height of more than 100 micrometres) [30,31].
2. Larger area fabrication (for application such as mammography, e.g., for a field of view of 10 × 10 cm${}^{2}$ or 15 × 15 cm${}^{2}$).
3. Acceptable uniformity across the absorption masks (no distortions and changes in the period and height).
4. Two-dimensional high-aspect ratio structures (2D gratings) for sensitivity in the *x* and *y* direction.

**Table 1:** List of today’s EI-XPCi systems with source, setup size (source to detector), masks, and detector information.

Reference	X-ray Source, Energy or Potential	$z_{\mathrm{so}}+z_{\mathrm{od}}$	M1 Aperture, Period	M2 Aperture, Period	Detector, Pixel Pitch
2017 [32]	Rigaku MM007, 35 kV${}_{p}$	>160 cm	16 μm, 134 μm	20 μm, 167 μm	Anrad SMAM A-Se, 85 μm
2017 [33]	Rigaku MM007, 40 kV${}_{p}$	200 cm	12 μm, 48 μm	15 μm, 62 μm	Pixirad, 62 μm
2018 [34]	Hamamatsu L8121-03, 40 keV	184 cm	—	—	Medipix3RX, 55 μm
2019 [35]	Rigaku MultiMax-9, 40 kV${}_{p}$	300 cm	10 μm, 79 μm	17 μm, 98 μm	PixiRad-2, 62 μm
2020 [36]	Rigaku MM007, 35 kV${}_{p}$	245 cm	10 μm, 79 μm	17 μm, 98 μm	Hamamatsu C9732DK-11, 100 μm ^1^
2022 [37]	Rigaku MM007, 40 kV${}_{p}$	100 cm	-	20 μm, 98 μm	Hamamatsu C9732DK, 50 μm

^1^ Effective pixel size.

The problem becomes even more challenging when the energy of the X-ray source increases, where, in order to stop the incident X-ray and to translate the X-ray to smaller beamlets at absorption masks, either the absorption part in the masks should be selected from materials with a higher atomic number (harder materials for the range of X-ray energy of interest), or the thickness of the absorption part should increase.

It should be noted that, to address these bottlenecks from the technological perspective, we are both limited to a few number of X-ray stopper materials, the most common X-ray absorber being used in fabrication technology is gold (Au), and the fabrication technology for high-resolution high-aspect ratio absorption masks is not as developed and available as what it is for high-resolution detectors. For instance, in the case of employing a high-resolution detector (7.8 μm pixel pitch) in an EI-XPCi setup, an almost 100 μm thick gold is required to stop 90% of a 40 keV X-ray; considering a compact system with $z_{so}$ = 18 cm and $z_{od}$ = 8 cm, it requires a pre-sample mask with an aperture size of less than 2 μm and period of less than 7 μm—the same condition with slightly bigger aperture size and 7.8 μm period applies for a detector mask; not to mention that by increasing distances, these values should decrease to match the EI-XPCi system requirement. This implies the importance of putting more effort into improving X-ray absorption mask fabrication technology for high-resolution detectors and high-energy X-ray imaging applications.

Amongst different techniques developed for X-ray absorption mask fabrication, there are two well-defined and widely used processes: etching processes—such as deep reactive ion etching (DRIE) on a silicon wafer [38]—and making molds on a conductive substrate, both followed by an electroplating step to create the absorber parts. The former relies on tools such as atomic layer deposition (ALD) of metals and DRIE process, which impede this fabrication method from scaling up and creating larger X-ray masks. The latter approach of making X-ray masks, however, is based on a mechanically stable mold (the most common one widely used is the negative photoresist SU-8) and a lithography process, either X-ray or UV lithography [39,40]. Although X-ray lithography is an efficient tool for high-aspect ratio structure fabrication [41,42,43,44], it is an expensive and hard-to-access method. On the other hand, employing conventional UV lithography techniques with SU-8 photoresist, in the best-case scenario, results in structures with aspect ratios as high as 10—which is not enough for a micro-scale pixel pitch high-resolution EI-XPCi system such as BrillianSe™ X-ray detector. Having said that, we could not experimentally test our high-resolution X-ray detector in an EI-XPCi configuration because of not having access to compatible absorption masks. Although a single-mask EI-XPCi method relaxes the conditions in the EI-XPCi systems, it still requires an absorption mask compatible with BrillianSe™ X-ray detector. We, however, tested a single-mask EI-XPCi system using an absorption mask with greater aperture size and period—employing a BrillianSe™ prototype—and its preliminary results are the subject of forthcoming publications. Employing a micron-scale pixel pitch X-ray detector in a single-mask EI-XPCi setup opens up new opportunities and possibilities to perform imaging tasks with less complexity and at lower dose exposures. Regarding the high-aspect ratio gratings, we are developing a new fabrication process to realize high-aspect ratio high-resolution absorption gratings using SU-8 photoresist and the UV LIGA process. The early results of this fabrication process are published in different works [45,46,47].

## 5. Conclusions

In this research, we investigated PB-XPCi and EI-XPCi techniques in terms of their contrast, edge-enhancement, visibility, and dose efficiency characteristics for a direct conversion detector with micron-scale pixel pitch. We used numerical modelling and a simulation tool to evaluate both techniques and demonstrate the effect of absorption mask thickness and environmental vibration on the performance of the EI-XPCi system. The numerical modelling was verified by employing a direct conversion amorphous selenium (a-Se)/CMOS based micron-scale pixel X-ray detector, BrillianSe™, in the PB-XPCi configuration to highlight the potential of such a detector in revealing small feature size structures. Although the EI-XPCi configuration demonstrated better performance in simulation results, there are some experimental challenges associated with using such a high-resolution detector. Two main challenges in a high-resolution EP-XPCi system studied in this work are the fabrication of high-resolution high-aspect ratio X-ray absorption gratings and the effect of mechanical vibrations on the system performance. Although the latter challenge can be addressed by using higher precision mechanical stages to dampen the mechanical vibrations, we believe that, by overcoming the challenges in making large-area X-ray grating technology, EI-XPCi systems can be translated into clinical imaging for applications where accuracy and dose efficiency are of importance, such as mammography.

## Data Availability

Not applicable.

## References

[1] Endrizzi M. (2018). X-ray phase-contrast imaging. Nucl. Instrum. Methods Phys. Res. Sect. A Accel. Spectrometers Detect. Assoc. Equip..

[2] Taba S.T., Gureyev T.E., Alakhras M., Lewis S., Lockie D., Brennan P.C. (2018). X-ray phase-contrast technology in breast imaging: Principles, options, and clinical application. Am. J. Roentgenol..

[3] Bravin A., Coan P., Suortti P. (2012). X-ray phase-contrast imaging: From pre-clinical applications towards clinics. Phys. Med. Biol..

[4] Wilkins S., Gureyev T.E., Gao D., Pogany A., Stevenson A. (1996). Phase-contrast imaging using polychromatic hard X-rays. Nature.

[5] Olivo A., Speller R. (2007). A coded-aperture technique allowing x-ray phase contrast imaging with conventional sources. Appl. Phys. Lett..

[6] Marenzana M., Hagen C.K., Borges P.D.N., Endrizzi M., Szafraniec M.B., Ignatyev K., Olivo A. (2012). Visualization of small lesions in rat cartilage by means of laboratory-based X-ray phase contrast imaging. Phys. Med. Biol..

[7] Lohr R.L., Scott C.C., Pil-Ali A., Karim K.S. (2020). A comparison of phase retrieval methods for propagation-based contrast X-ray imaging with polychromatic sources. Medical Imaging 2020: Physics of Medical Imaging.

[8] Burvall A., Lundström U., Takman P.A., Larsson D.H., Hertz H.M. (2011). Phase retrieval in X-ray phase-contrast imaging suitable for tomography. Opt. Express.

[9] Withers P.J., Bouman C., Carmignato S., Cnudde V., Grimaldi D., Hagen C.K., Maire E., Manley M., Du Plessis A., Stock S.R. (2021). X-ray computed tomography. Nat. Rev. Methods Prim..

[10] Lohse L.M., Robisch A.L., Töpperwien M., Maretzke S., Krenkel M., Hagemann J., Salditt T. (2020). A phase-retrieval toolbox for X-ray holography and tomography. J. Synchrotron Radiat..

[11] Olivo A., Speller R. (2008). Image formation principles in coded-aperture based X-ray phase contrast imaging. Phys. Med. Biol..

[12] Karim K.S., Scott C.C. (2019). Method and System for High-Resolution X-ray Detection for Phase Contrast X-ray Imaging. U.S. Patent.

[13] Koch A., Raven C., Spanne P., Snigirev A. (1998). X-ray imaging with submicrometer resolution employing transparent luminescent screens. JOSA A.

[14] Martin T., Koch A. (2006). Recent developments in X-ray imaging with micrometer spatial resolution. J. Synchrotron Radiat..

[15] Bech M., Bunk O., David C., Kraft P., Brönnimann C., Eikenberry E., Pfeiffer F. (2008). X-ray imaging with the PILATUS 100k detector. Appl. Radiat. Isot..

[16] Kasap S., Frey J.B., Belev G., Tousignant O., Mani H., Laperriere L., Reznik A., Rowlands J.A. (2009). Amorphous selenium and its alloys from early xeroradiography to high resolution X-ray image detectors and ultrasensitive imaging tubes. Phys. Status Solidi.

[17] Bidola P., Morgan K., Willner M., Fehringer A., Allner S., Prade F., Pfeiffer F., Achterhold K. (2017). Application of sensitive, high-resolution imaging at a commercial lab-based X-ray micro-CT system using propagation-based phase retrieval. J. Microsc..

[18] Cochard H., Delzon S., Badel E. (2015). X-ray microtomography (micro-CT): A reference technology for high-resolution quantification of xylem embolism in trees. Plant Cell Environ..

[19] Fonseca M.d.C., Araujo B.H.S., Dias C.S.B., Archilha N.L., Neto D.P.A., Cavalheiro E., Westfahl H., da Silva A.J.R., Franchini K.G. (2018). High-resolution synchrotron-based X-ray microtomography as a tool to unveil the three-dimensional neuronal architecture of the brain. Sci. Rep..

[20] Parsafar A., Scott C.C., El-Falou A., Levine P.M., Karim K.S. (2015). Direct-Conversion CMOS X-ray Imager With 5.6 *μ*m × 6.25 *μ*m Pixels. IEEE Electron Device Lett..

[21] Scott C.C., Abbaszadeh S., Ghanbarzadeh S., Allan G., Farrier M., Cunningham I.A., Karim K.S. (2014). Amorphous selenium direct detection CMOS digital X-ray imager with 25 micron pixel pitch. Medical Imaging 2014: Physics of Medical Imaging.

[22] Scott C.C., Farrier M., Li Y., Laxer S., Ravi P., Kenesei P., Wojcik M.J., Miceli A., Karim K.S. (2021). High-energy micrometre-scale pixel direct conversion X-ray detector. J. Synchrotron Radiat..

[23] Millard T., Endrizzi M., Ignatyev K., Hagen C., Munro P., Speller R., Olivo A. (2013). Method for automatization of the alignment of a laboratory based X-ray phase contrast edge illumination system. Rev. Sci. Instrum..

[24] Scott C. (2019). Hybrid Semiconductor Detectors for High Spatial Resolution Phase-Contrast X-ray Imaging. Ph.D. Thesis.

[25] Vittoria F.A., Diemoz P.C., Endrizzi M., Rigon L., Lopez F.C., Dreossi D., Munro P.R., Olivo A. (2013). Strategies for efficient and fast wave optics simulation of coded-aperture and other X-ray phase-contrast imaging methods. Appl. Opt..

[26] Lundström U. (2014). Phase-Contrast X-ray Carbon Dioxide Angiography. Ph.D. Thesis.

[27] Siewerdsen J., Waese A., Moseley D., Richard S., Jaffray D. (2004). Spektr: A computational tool for X-ray spectral analysis and imaging system optimization. Med. Phys..

[28] Palermo F., Pieroni N., Maugeri L., Provinciali G.B., Sanna A., Massimi L., Catalano M., Olbinado M.P., Bukreeva I., Fratini M. (2020). X-ray Phase Contrast Tomography Serves Preclinical Investigation of Neurodegenerative Diseases. Front. Neurosci..

[29] Karim K.S., Scott C.C. (2021). Method and System for High-Resolution X-ray Detection for Phase Contrast X-ray Imaging. U.S. Patent.

[30] Romano L., Vila-Comamala J., Schift H., Stampanoni M., Jefimovs K. (2017). Hot embossing of Au-and Pb-based alloys for X-ray grating fabrication. J. Vac. Sci. Technol. B Nanotechnol. Microelectron. Mater. Process. Meas. Phenom..

[31] Romano L., Vila-Comamala J., Kagias M., Vogelsang K., Schift H., Stampanoni M., Jefimovs K. (2017). High aspect ratio metal microcasting by hot embossing for X-ray optics fabrication. Microelectron. Eng..

[32] Kallon G., Diemoz P., Vittoria F., Basta D., Endrizzi M., Olivo A. (2017). Comparing signal intensity and refraction sensitivity of double and single mask edge illumination lab-based X-ray phase contrast imaging set-ups. J. Phys. D Appl. Phys..

[33] Zamir A., Diemoz P.C., Vittoria F.A., Hagen C.K., Endrizzi M., Olivo A. (2017). Edge illumination X-ray phase tomography of multi-material samples using a single-image phase retrieval algorithm. Opt. Express.

[34] Vespucci S., Lewis C., Park C.S., Das M. (2018). Examining phase contrast sensitivity to signal location and tissue thickness in breast imaging. Medical Imaging 2018: Physics of Medical Imaging.

[35] Kallon G., Vittoria F., Buchanan I., Endrizzi M., Olivo A. (2020). An experimental approach to optimising refraction sensitivity for lab-based edge illumination phase contrast set-ups. J. Phys. D Appl. Phys..

[36] Havariyoun G., Vittoria F.A., Hagen C.K., Basta D., Kallon G.K., Endrizzi M., Massimi L., Munro P., Hawker S., Smit B. (2019). A compact system for intraoperative specimen imaging based on edge illumination X-ray phase contrast. Phys. Med. Biol..

[37] Massimi L., Kallon G.K., Buchanan I., Endrizzi M., Dobrosz P., Brooks R., Brau D., Bullard E., Olivo A. (2022). Replacing the detector mask with a structured scintillator in edge-illumination X-ray phase contrast imaging. J. Appl. Phys..

[38] Kagias M., Wang Z., Guzenko V.A., David C., Stampanoni M., Jefimovs K. (2019). Fabrication of Au gratings by seedless electroplating for X-ray grating interferometry. Mater. Sci. Semicond. Process..

[39] Noda D., Tanaka M., Shimada K., Yashiro W., Momose A., Hattori T. (2008). Fabrication of large area diffraction grating using LIGA process. Microsyst. Technol..

[40] Mohr J., Grund T., Kunka D., Kenntner J., Leuthold J., Meiser J., Schulz J., Walter M. (2012). High aspect ratio gratings for X-ray phase contrast imaging. AIP Conference Proceedings.

[41] Ehrfeld W., Lehr H. (1995). Deep X-ray lithography for the production of three-dimensional microstructures from metals, polymers and ceramics. Radiat. Phys. Chem..

[42] Guckel H. (1998). High-aspect-ratio micromachining via deep X-ray lithography. Proc. IEEE.

[43] Kenntner J., Altapova V., Grund T., Pantenburg F.J., Meiser J., Baumbach T., Mohr J. (2012). Fabrication and characterization of analyzer gratings with high aspect ratios for phase contrast imaging using a Talbot interferometer. AIP Conference Proceedings.

[44] David C., Bruder J., Rohbeck T., Grünzweig C., Kottler C., Diaz A., Bunk O., Pfeiffer F. (2007). Fabrication of diffraction gratings for hard X-ray phase contrast imaging. Microelectron. Eng..

[45] Pil-Ali A., Soltani M., Adnani S., Cui B., Karim K.S. (2022). A novel multi-layer grating structure for X-ray phase-contrast imaging. Medical Imaging 2022: Physics of Medical Imaging.

[46] Pil-Ali A., Soltani M., Adnani S., Kayaharman M., Poudineh M., Cui B., Karim K.S. (2022). Improving adhesion quality of SU-8 to gold thin film for absorption grating fabrication in X-ray phase-contrast imaging. Medical Imaging 2022: Physics of Medical Imaging.

[47] Pil-Ali A., Adnani S., Gavirneni P., Shin S., Sadeghimakki B., Poudineh M., Wong W., Karim K.S. (2022). Self-aligned fabrication of high-aspect ratio high-resolution X-ray gratings. Medical Imaging 2022: Physics of Medical Imaging.

